# Hotspots of recent hybridization between pigs and wild boars in Europe

**DOI:** 10.1038/s41598-018-35865-8

**Published:** 2018-11-26

**Authors:** Laura Iacolina, Cino Pertoldi, Marcel Amills, Szilvia Kusza, Hendrik-Jan Megens, Valentin Adrian Bâlteanu, Jana Bakan, Vlatka Cubric-Curik, Ragne Oja, Urmas Saarma, Massimo Scandura, Nikica Šprem, Astrid Vik Stronen

**Affiliations:** 1 Department of Chemistry and Bioscience, Aalborg University, Frederik Bajers Vej 7H, 9220 Aalborg, Denmark; 2 Aalborg Zoo, Mølleparkvej 63, 9000 Aalborg, Denmark; 3 Centre for Research in Agricultural Genomics (CRAG), CSIC-IRTA-UAB-UB, Campus de la Universitat Autònoma de Barcelona, Bellaterra, 08193 Spain; 4 Departament de Ciència Animal i dels Aliments, Universitat Autònoma de Barcelona, Bellaterra, 08193 Spain; 5 Animal Genetics Laboratory, Faculty of Agricultural and Food Sciences and Environmental Management, University of Debrecen, Böszörményi 138, 4032 Debrecen, Hungary; 6 Wageningen University & Research, Animal Breeding and Genomics, Droevendaalsesteeg 1, Wageningen, 6708PD The Netherlands; 7 Institute of Life Sciences, Faculty of Animal Science and Biotechnologies, University of Agricultural Sciences and Veterinary Medicine, Calea Mănăştur 3-5, 400372 Cluj-Napoca, Romania; 8 Technical University of Zvolen, Department of Phytology, Ul. T. G. Masaryka 24, 96053 Zvolen, Slovakia; 9 Department of Animal Science, Faculty of Agriculture, University of Zagreb, Svetošimunska cesta 25, 10000 Zagreb, Croatia; 10 Department of Zoology, Institute of Ecology and Earth Sciences, University of Tartu, Vanemuise 46, 51003 Tartu, Estonia; 11 Department of Veterinary Medicine, University of Sassari, via Muroni 25, I-07100 Sassari, Italy; 12 Department of Fisheries, Beekeeping, Game Management and Special Zoology, Faculty of Agriculture, University of Zagreb, Svetošimunska cesta 25, 10000 Zagreb, Croatia; 13 Department of Biology, Biotechnical Faculty, University of Ljubljana, Večna pot 111, 1000 Ljubljana, Slovenia

**Keywords:** Genomics, Population genetics

## Abstract

After a strong demographic decline before World War II, wild boar populations are expanding and the species is now the second-most abundant ungulate in Europe. This increase raises concerns due to wild boar impact on crops and natural ecosystems and as potential vector of diseases. Additionally, wild boar can hybridize with domestic pigs, which could increase health risks and alter wild boar adaptive potential. We analysed 47,148 Single Nucleotide Polymorphisms in wild boar from Europe (292) and the Near East (16), and commercial (44) and local (255) pig breeds, to discern patterns of hybridization across Europe. We identified 33 wild boars with more than 10% domestic ancestry in their genome, mostly concentrated in Austria, Bosnia and Herzegovina, Bulgaria and Serbia. This difference is probably due to contrasting practices, with free-ranging *vs*. industrial farming but more samples would be needed to investigate larger geographic patterns. Our results suggest hybridization has occurred over a long period and is still ongoing, as we observed recent hybrids. Although wild and domestic populations have maintained their genetic distinctiveness, potential health threats raise concerns and require implementation of management actions and farming practices aimed at reducing contact between wild and domestic pigs.

## Introduction

Wild boar (*Sus scrofa*) populations underwent severe demographic declines till the middle of the 20^th^ century in certain areas of Europe (e.g. Denmark, Sweden, Italy), but they are currently expanding at a fast pace due to a variety of favourable factors such as the relatively low number of predators, climate change, intensification of crop production, supplementary feeding, reforestation of agricultural areas, decrease in hunting pressure, compensatory population responses to hunting and intentional releases for hunting purposes^1–4^. Today, the wild boar is the second-most abundant ungulate in Europe, with nearly four million individuals, and is considered a pest in many areas due to crop damage, ecological impact on other species and road collisions^5–7^. Furthermore, this expansion is considered a threat to wildlife (particularly ground nesting birds^8^), livestock, and human health^9,10^.

Hybridization between domestic pig (*Sus scrofa domesticus*, henceforth pig) and wild boar seems to have been quite pervasive since domestication^11^. The approximate Bayesian computation analysis of 103 genomes of Asian and European wild boar and domestic pigs demonstrated the existence of gene flow during and after domestication^12^. Indeed, the keeping of pigs in enclosures and sties is relatively recent and coincides with the intensification of pig production that began in England during the 17^th^-18^th^ centuries^13^. Even nowadays, and despite swine production being mostly carried out indoors and at an industrial scale, gene flow between wild boar and pigs appears to be quite frequent^14^. Goedbloed *et al*.^15^ genotyped, with the Porcine SNP60 Beadchip^16^, 88 wild boar from northwest Europe and observed that 10% of individuals harboured an excess of rare Single Nucleotide Polymorphisms (SNP) compatible with recent introgression (estimated to be first to fifth generation backcrosses) from multiple domestic sources. In North-West Europe, the main source of introgression of pigs into wild boar appears to come from released or escaped farmed wild boar – a misnomer since many farmed wild boar are hybrids that are easier to rear and grow faster than pure wild boar^17^. Similar results have been obtained in genome-wide analyses of the variation of other wild boar populations from Sardinia^18^ and Romania^19^, where introgression of pigs into wild boar may be related to free-ranging pig farming.

Although the introgression of domestic genes might, in some cases, cause outbreeding depression and maladaptation to the environment^20^, admixed genotypes could potentially adapt better than their parental populations^21^, a hybrid vigour that might be due to increased heterozygosity^22^, and display higher reproductive rates^6,23^, thus augmenting the invasiveness potential of this species^21^. Furthermore, genetic fitness and long-term viability of pure wild boar populations could be threatened by the spread of infectious diseases and the competition with hybrids for environmental resources. The main goal of our study was to generate an overall picture of the levels of recent porcine introgression in European wild boar by analysing the genome-wide diversity of specimens with a broad geographic distribution. We were also interested in determining if the frequencies of hybrids appear to be homogeneous across European countries or if, on the contrary, there are geographic differences, such as hotspots or coldspots of hybridization. Even in cases where the same genotyping platform is used, the number of sampled individuals and the analytical approach can differ greatly amongst studies, thus making difficult to compare the corresponding hybridization rate estimates. This unresolved issue is of particular relevance in the current European situation, where interaction between the two forms may pose sanitary threats.

## Materials and Methods

### Sampling and genotyping

**Figure 1:**
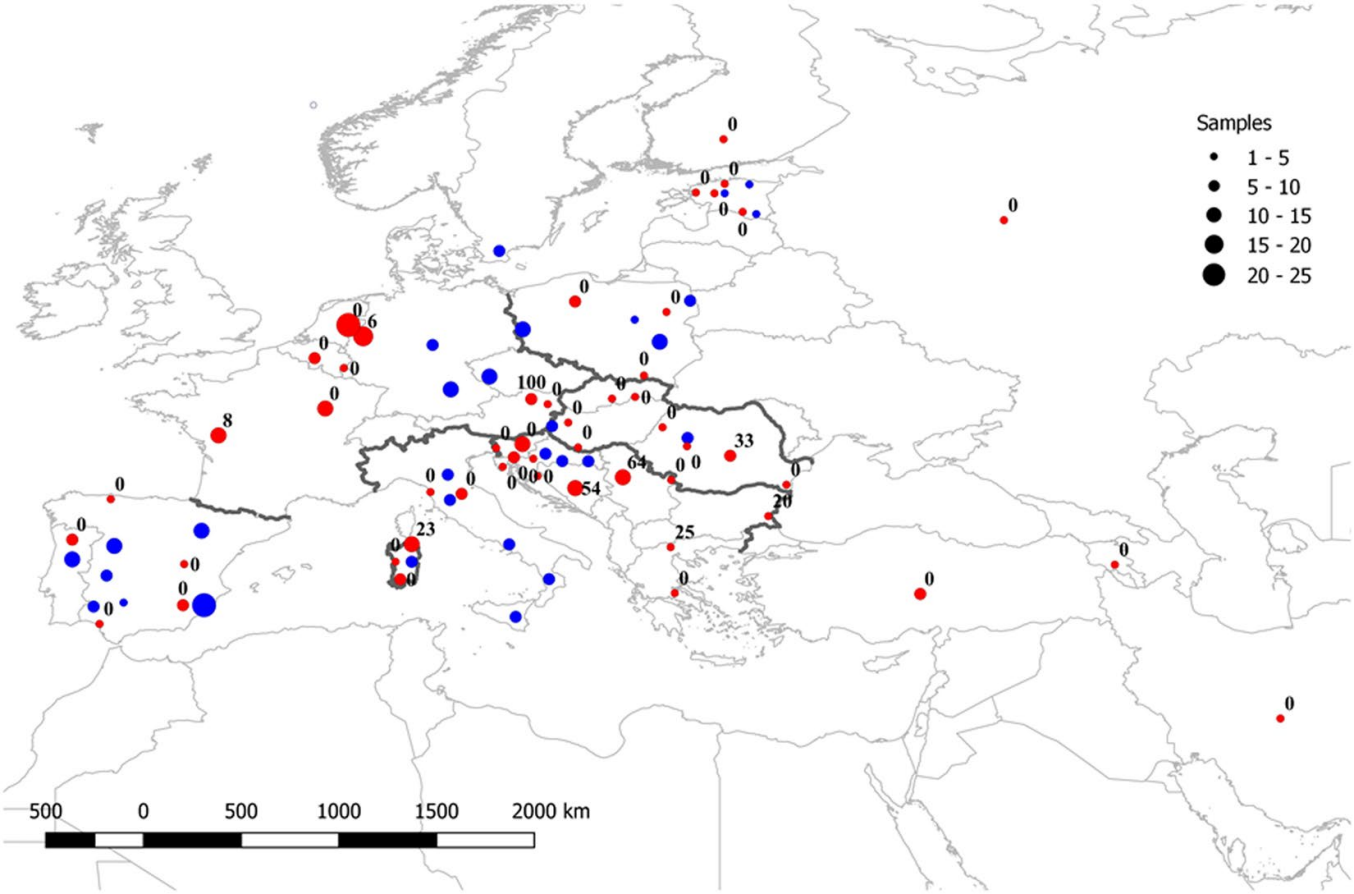
Map of Europe with sampling locations and percentage of detected hybrids in the wild boar population. Circles are proportional to sample size, wild boar are in red () and domestic pig in blue ().

Samples from 82 European wild boars (Austria, Bosnia and Herzegovina, Estonia, Hungary, Poland, Serbia and Slovakia) and 60 domestic pigs (Croatia, Estonia and Poland) were provided by local hunters and veterinarians and collected according to National laws, no animal was specifically killed for this research. These 142 individuals were genotyped with the Porcine SNP60 Beadchip^16^, according to the manufacturer’s instructions (http://www.illumina.com/products/porcineSNP60_dna_analysis_kit.ilmn) at GenoSkan A/S (Denmark). The resulting 60 K genotypes were merged with publicly available data from Near Eastern (N = 19) and European (N = 334) wild boar and domestic pigs (N = 318)^18,19,24^. The dataset was analysed with PLINK 1.9^25^ for filtering according to quality (call rate >0.9, missing genotypes <10%) and relatedness (identity by descent) criteria. Whenever possible, without compromising the sample size, we removed one individual from closely related pairs showing a high degree of relatedness (first order relatives). Additionally, as unequal sample size could potentially bias the estimates of diversity measures, levels of differentiation and cluster inference, the number of individuals in each population was equalized. We randomly removed, in R 3.5.0^26^, individuals from large populations to obtain a maximum sample size of 25. The resulting pruned dataset consisted of 16 Near Eastern and 290 European wild boar and 299 pigs belonging to five international (N = 44) and 22 local breeds (N = 255), genotyped at 47,148 (47 K) autosomal loci (Fig. 1 and Table 1; for additional details see Table S1).

**Table 1:** Variability levels in the analysed clusters.

Cluster	Sample size	N of polymorphic loci	H_e_	H_o_	MAF (±SD)
DP Commercial	44	44104	0.344	0.269	0.262 (±0.147)
DP Balkans	23	39258	0.255	0.231	0.186 (±0.155)
DP Carpathians	25	40137	0.261	0.217	0.194 (±0.160)
DP Central-East Europe	19	40766	0.310	0.321	0.235 (±0.158)
DP Central-Nord Europe	22	43464	0.332	0.333	0.253 (±0.152)
DP Central-West Europe	35	43441	0.327	0.313	0.248 (±0.151)
DP Iberia	83	43804	0.294	0.242	0.218 (±0.153)
DP Italy	48	43612	0.304	0.238	0.227 (±0.152)
WB Near East	16	28575	0.186	0.172	0.136 (±0.157)
WB Balkans	67	36518	0.231	0.212	0.174 (±0.167)
WB Carpathians	37	37292	0.232	0.210	0.174 (±0.165)
WB Central-North-Eastern Europe	34	31758	0.225	0.215	0.169 (±0.168)
WB Central-West Europe	85	39978	0.235	0.197	0.176 (±0.166)
WB Iberia	23	29508	0.214	0.189	0.161 (±0.168)
WB Italy	19	29107	0.206	0.177	0.153 (±0.165)
WB Sardinia	25	31669	0.191	0.165	0.141 (±0.160)

Cluster = group of wild boar (WB) or domestic pig (DP) considered; Sample size = number of individuals, Polymorphic loci = number of polymorphic loci, H_e_ = expected heterozygosity, H_o_ = observed heterozygosity, MAF = minor allele frequency, SD = standard deviation.

### Statistical analysis

To determine the amount of genetic differentiation among populations, we performed a Principal Component Analysis (PCA) with adegenet^27^ in R. To avoid the potential confounding effect of the high divergence between wild boar and domestic pigs, we did a second analysis of European wild boar that included only 25 randomly selected domestic pigs as a reference. The aim of this latter analysis was to investigate the specific structure of wild boar populations. Based on the PCA results, geographic information and previous findings^18^, we grouped the wild boar and domestic pig populations into eight clusters each: wild boar from: (1) Near East, (2) Balkans, (3) Carpathians, (4) Iberia, (5) Italy (mainland), (6) Sardinia, (7) Central-West Europe (WB-CW), (8) Central-North-Eastern Europe (WB-CNE); domestic pigs from: (1) Balkans, (2) Carpathians, (3) Central-East Europe (DP-CE), (4) Central-North Europe (DP-CN), (5) Central-West Europe (DP-CW), (6) Iberia, (7) Italy and (8) commercial breeds (see Table 2 for countries included in each cluster). Variability levels of the populations were assessed by computing minor allele frequencies (MAF, which indicates the abundance of rare alleles through the genome), as well as expected (H_e_) and observed (H_o_) heterozygosities (which are measurements of genetic variability) within clusters with PLINK. Genetic differentiation among populations was estimated by calculating pairwise F_ST_ values with Arlequin 3.5^28^. For this analysis, loci in linkage disequilibrium (r^2^ > 0.5) were removed with PLINK, to reduce bias due to physical linkage between loci^29^, resulting in a reduced dataset of 29,802 (30 K) SNPs.

**Table 2:** Number and percentage of hybrids for each analysed population.

Wild boar Population	Population abbreviation	Sample size	N hybrids	% hybrids
Bosnia and Herzegovina	WBos	13	7	53.8%
Bulgaria	WBul	5	1	20.0%
Croatia	WCro	15	0	0.0%
Greece	WGre	8	1	12.5%
Serbia	WSer	14	9	64.3%
Slovenia	WSlv	12	0	0.0%
**Balkans**		**67**	**18**	**26.9%**
Hungary	WHun	14	0	0.0%
Romania	WRom	18	2	11.1%
Slovakia	WSlk	5	0	0.0%
**Carpathians**		**37**	**2**	**5.4%**
Portugal	WPor	9	0	0.0%
Spain	WSpa	14	0	0.0%
**Iberia**		**23**	**0**	**0.0%**
**Italy**	**WIta**	**19**	**0**	**0.0%**
**Sardinia**	**WSar**	**25**	**3**	**12.0%**
Austria	WAus	9	8	88.9%
Belgium	WBel	6	0	0.0%
France	WFra	25	1	4.0%
Germany	WGer	16	1	6.3%
Luxembourg	WLux	4	0	0.0%
Netherlands	WNed	25	0	0.0%
**Central-West Europe**		**85**	**10**	**11.8%**
Estonia	WEst	15	0	0.0%
Finland	WFin	3	0	0.0%
Poland	WPol	12	0	0.0%
Russia	WRus	4	0	0.0%
**Central-North-Eastern Europe**		**34**	**0**	**0.0%**
**TOT**		**290**	**33**	**11.4%**

N hybrids = number of hybrids in the population, % hybrids = percentage of hybrids in the population.

We performed an initial assessment of population structure based on the 30 K dataset and all individuals with the maximum likelihood approach implemented in Admixture v1.23^30^. Default settings plus a bootstrap of 1000 and a cross-validation of 10 for values of K from 1 to 30 were used in this analysis. The most likely number of populations was determined based on the lowest cross-validation error^30^. To assess introgression levels, we subsequently performed the Admixture analysis independently for each geographic area (Balkans, Carpathians, Central Europe, Iberia, mainland Italy, Sardinia, Northern Europe). For this purpose, we used all the wild boar and domestic pig samples from the geographic area under consideration as well as wild boar from neighbouring countries and commercial breeds. In these analyses we used the same parameters previously described and the maximum K values listed in Table S2. Hybrids were identified when >10% of their genome had domestic ancestry. We chose this threshold because the average wild boar-ancestry across wild boar populations was >90%. For populations that were not represented by a single cluster this value was computed by summing values of all the wild boar-clusters present in the population.

Furthermore, we repeated the PCA and Admixture analyses with the same samples and parameters previously employed (see Table S2 for maximum K values), by considering the 983 (henceforth 1 K) most informative SNPs to distinguish between wild boar and pigs based on the initial PCA loadingplot values. Hybrid identification threshold was the same as above (10%). We identified additional candidate hybrids based on the 47 K and 1 K PCA results.

We evaluated the proportion of hybrid individuals within a country based on the number of individuals that were identified as admixed in at least 30% of the analyses. Comparisons focusing on the relevant geographic area (e.g. the Netherlands and neighbouring states) were weighted 100%, whereas those focusing on other areas or on the whole sample were considered 80% for this calculation.

## Results

### Population characterization and variability

**Figure 2:**
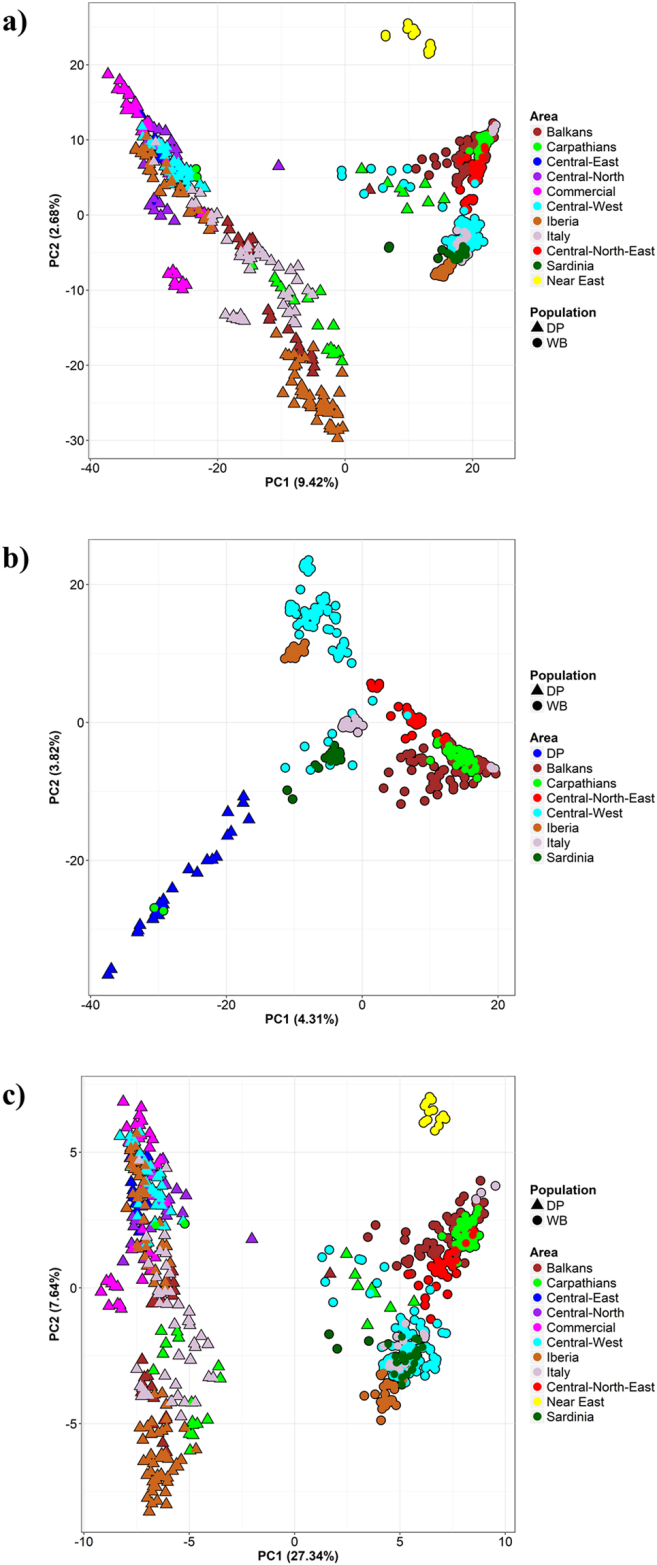
Principal Component Analysis of wild boar (WB) and domestic pig (DP) whole genome genotypes. (**a**) Entire dataset based on 47 K SNP; (**b**) Reduced dataset with all European wild boar and only 25 pigs as reference, based on 47 K SNP; (**c**) Reduced dataset with all wild boar and pigs, based on 1 K SNP.

In the PCA based on 47 K SNPs and 607 individuals, PC1 splits wild boar and pig populations, and PC2 separates European and Near Eastern wild boar (Fig. 2a). The marked wild boar-domestic pig divergence on PC1 makes it difficult to visualize the population structure in European wild boar, so we repeated the PCA with all European wild boar and 25 pigs as reference (Fig. 2b). In the resulting plot, PC1 shows a wild boar-domestic pig split and a moderate north-south gradient within wild boar, whereas we observe an east-west gradient within wild boar along PC2 (Fig. 1b). In the PCA based on 1 K SNPs, PC1 reveals a sharp wild boar-domestic pig division (Fig. 2c), with PC2 separating European and Near Eastern wild boar in a pattern concordant with Fig. 2a.

The number of polymorphic loci ranged from 28,575 (60.6%) in Near Eastern wild boar to 44,104 (93.5%) in commercial pigs, Near Eastern wild boar (0.136) and commercial pigs (0.262) also represent the two extremes of the MAF range (Table 1). With the exception of DP-CE and DP-CN all populations showed higher H_e_ than H_o_, with H_e_ ranging from 0.186 (Near Eastern wild boar) to 0.344 (Commercial breeds) and H_o_ in the range 0.165 (Sardinian wild boar) − 0.333 (DP-CN) (Table 1). Pairwise F_st_ values, calculated on the 30 K SNPs dataset, were lower among domestic breeds than among wild boar populations (Mann–Whitney U test, t: 2.715, p: 0.009). Values ranged from 0.045 (commercial breeds– DP-CW) to 0.152 (Balkan breeds– DP-CE), whereas the most divergent population was Near Eastern wild boar (range 0.186–0.307, compared to Balkan and Sardinian wild boar respectively). The magnitude of differentiation between European wild boar and domestic pigs was quite variable, with a minimum of 0.090 between WB-CW and Carpathian breeds and a maximum between Sardinian wild boar and DP-CE (0.241). Among European wild boar populations, the least divergent ones were Carpathians and Balkans (0.018) whereas the most divergent populations were Sardinian and Iberian (Table S3).

### Identification of hybrids

**Figure 3:**
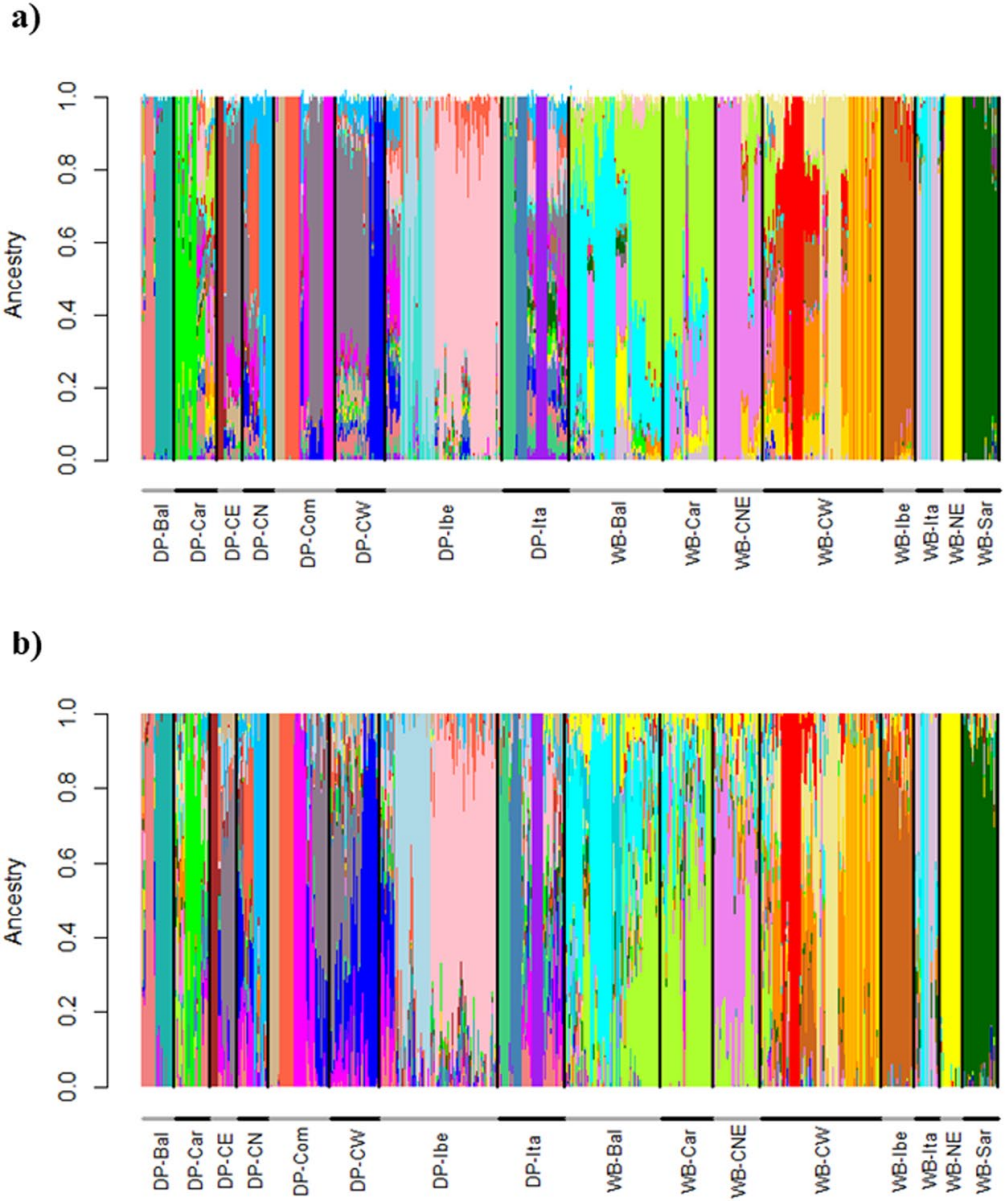
Admixture plots of European wild boar and domestic pigs. Plots represent all wild boar and pigs (K = 27) based on 30 K SNP (**a**) and 1 K SNP (**b**). Domestic Pig: DP-Com = commercial breeds; DP-CN = Central-Nord Europe; DP-CE = Central-East Europe; DP-CW = Central-West Europe; DP-Ita = Italy; DP-Ibe = Iberia; DP-Car = Carpathians; DP-Bal = Balkans; wild boar: WB-Sar = Sardinia; WB-Ita = Italy; WB-CW = Central-West Europe; WB-CNE = Central-North-Eastern Europe; WB-Ibe = Iberia; WB-Car = Carpathians; WB-Bal = Balkans; WB-NE = Near Eastern.

Admixture analyses were initially based on all the available samples and the 30 K dataset. The cross-validation error showed a decreasing tendency without reaching a plateau, with a first levelling off at K = 27. Such results highlight the complexity of both the wild and domestic populations (Fig. 3a). The observed substructure could be an important tool for animal traceability, allowing the monitoring of natural expansion - like in the observed case of German wild boar with French or WB-CNE ancestry, as well as Balkan wild boar among the Italian sample – and, potentially, the identification of translocated individuals. The analyses by geographic area allowed us to infer the contributions of local breeds to the hybridization events and evaluate the relationships among populations at a finer geographic scale (Figs S1,S3). For example, we observed the presence of multiple clusters at the within-country level (e.g.: France, the Netherlands), but also areas showing a gradient among clusters (e.g. Balkans, Carpathians). This, incidentally, also highlights the importance of including references from as many sources of gene-flow as possible, as applied in our study (Fig. S1). Such a strategy is particularly helpful in characterizing population substructure within both wild and domestic populations, as earlier suggested by Steyer *et al*.^31^.

The analysis with Admixture of the 1 K dataset was highly congruent with the 30 K dataset for K values between 2 and 4. At K = 2 wild boar were roughly separated from pigs although most populations, including commercial breeds, show high levels of introgression. At K = 3, the dataset was divided in wild boar, local and commercial pig breeds, while at K = 4 the distinction was among Western European, Near Eastern plus Balkan-Carpathian wild boar, local and commercial breeds (Fig. S2). However, most populations had genomic contributions from more than one cluster, resulting in low power for hybrid identification. Because of that, we chose to focus on the most likely K-value identified with the cross-validation criterium for each analyses (see Table S2 for K-values). Concordantly, both sets exhibited intricate relationships among populations (Fig. 3). The fine scale geographic investigation reported above permitted us to identify the contribution of local pig breeds to hybridization events and determine the relationships among wild populations with a relatively high resolution (Figs S1,S3).

The average attribution to the overall wild boar cluster, across analyses, was the highest for WB-CNE (0.986) and lowest for Balkan wild boar (0.931) varying for local populations from the 0.999 of Slovakia to the 0.784 of Austria. The percentage of hybrids varied greatly among countries (0%-89%) with an average of 11.4% across Europe but being mostly concentrated in a few countries (Table 2). Using meridian 14°E as a reference, we observed 19% and 3.5% hybrids in Eastern (N = 147) and Western (N = 143) Europe, respectively. However, the higher detection rate in the east were primarily explained by the findings from Bosnia and Herzegovina, Serbia and Bulgaria.

## Discussion

Hybridization between wild boar and pigs has been detected in several European countries by using a variety of markers (e.g.^15,32,33^). Although providing valuable regional information, these studies cannot be compared in a straightforward manner, thus making it difficult to evaluate the extent of wild boar x domestic pig hybridization across Europe. The use of Porcine SNP60 Beadchip data allows us to easily integrate genotypic information from samples analysed in different laboratories^34^. As recently shown by Pilot *et al*.^35^ for wolves and dogs using 61 K SNPs, genome-wide information can substantially improve the precision with which the spatio-temporal levels of hybridization are quantified. Considering our aim was to assess the hybridization levels in wild boar across Europe we compared wild boar with commercial pigs, to control for the accuracy of our results as no hybridization was expected in industrially raised pigs. Additionally, we included local breeds as, in principle, they are expected be the main source of porcine introgression into wild boars, whereas industrially raised pigs would have a much less relevant role. Furthermore, as highlighted by both the complexity of our Admixture results (Fig. 3) and the within-cluster gradient in the PCA (Fig. 2), local breeds can display a strong genetic differentiation when compared to commercial lines and their inclusion is of paramount importance for the estimation of recent hybridization levels.

The incorporation in the dataset of multiple potential sources of hybridization, and the recent shared common ancestry between wild boar and pigs, makes the identification of admixed individuals challenging, as shown by the Admixture analyses at K = 2. An additional difficulty is to define non-admixed individuals i.e. any wild boar population may present some level of ancient or recent introgression from pigs. Conceivably, the group least likely to have experienced recent introgression is the commercial lines, as their breeding history is recorded. An assumption of no introgression is commonly required by most admixture analyses. However, Pilot *et al*.^35^ recently reported that the identification of hybrids, was not greatly affected by the analytical approach employed in their identification and the composition of the dataset. To account for potential biases, and in agreement with previous studies suggesting the importance of combining different approaches^36^, we chose to consider as hybrids only those individuals that were concordantly identified as such by multiple analyses. This led to the identification of an overall 11.4% level of hybridization across Europe, with high variability among countries (0–89%, Table 2). This result is congruent with previous studies that reported recent hybridization ranging from absent (Iberia, using mitochondrial DNA -mtDNA^37^) to highly prevalent (Ireland, using microsatellite and mtDNA^38^). It is interesting to notice that recent hybridization is particularly common in the Balkans and Carpathians, as well as in Sardinia, areas where free ranging farming is still commonly practiced^18,19,39^. Intriguingly, no introgressed individual has been detected in Croatia, where free-ranging farming was prohibited in 2007 to prevent Classical Swine Fever epidemics^40^. However, recent hybridization has been observed also in countries where industrial farming is dominant such as Austria, Germany and France. Such observations can be due to the introductions and/or escape of farmed individuals^17,41^. More specifically, two populations deserve particular attention as our results deviated from expectations: the Netherlands and Austria. Although we started with the dataset of Dutch individuals analysed by Goedbloed at al.^15^, where they reported 10% hybridization, we did not observe any signal of admixture. Several factors could have led to this lack of concordance. First of all, to equalize sample size across populations, we strongly reduced the number of analysed wild boar (from 88 to 25 individuals). This random selection may have left out introgressed individuals by chance. However, by repeating the analyses with different individuals we obtained the same results and we can thus rule out such an explanation. Furthermore, Goedbloed *et al*.^15^ focused their analyses on the identification of pig-specific alleles and used them, combined with simulations, to detect past (backcrosses up to the fifth generation) admixture events. This approach is extremely region specific and allows high resolution, but it is not suitable for performing investigations comprising individuals from multiple geographic areas. This important methodological difference may have been the reason of the observed differences between results. However, it is interesting to notice that they observed past admixture events mostly outside the two nature conservation areas where wild boar were introduced in the early 20^th^ century^5^. According to Dutch nature conservation legislation wild boar outside of these areas are culled. The main source of hybrids in the Netherlands therefore appears to be farm escapees or released individuals, which usually are killed before they can contribute to established wild boar populations, thus limiting the spread of introgressed genes.

The other unexpected result was the extremely high levels of porcine introgression recorded in Austria. As our approach can only detect recent admixture events^35^ a possible explanation could be a recent release of farmed wild boar, either legal or illegal, as farmed animals are more likely to show introgression from pigs^17^. If these released animals establish a self-sustaining population in an area where no native wild boar is present, the domestic contribution will remain high^32^. If such newly established populations subsequently expand their range, the pig contributions may spread at high frequencies in a broader contiguous region, and from there establish a source of pig variation which can be introgressed into neighbouring wild boar populations. An example of this was previously found by Goedbloed *et al*.^15^. However, we cannot exclude the possibility that individuals belonging to semi-feral breeds were included in our sample, as the so called “forest pigs”, which are both an attraction and source of income, are re-gaining popularity in some areas of Austria^42^. Further investigation of this Austrian population, covering a larger area of its present distribution and increasing the number of samples is thus needed to evaluate the degree of introgression in this country.

The levels of hybridization detected in our study (11.4%) are concordant with previous results found from analyses with a variety of markers^15,19,43^, confirming that hybridization with pigs has occurred in multiple locations across Europe. Ancestry levels between 0 and 0.25 would be expected in the presence of regular hybridization events over generations, with back-crosses and gene introgression, whereas recent (first or second generation) admixture events would lead to values between 0.25 and 0.5^35^. In our wild boar dataset we mostly observed pig ancestry ranging from 0 to 0.25, although we only considered as hybrids those wild boars with a domestic ancestry above 10%. Interestingly, we detected seven individuals with pig ancestry ranging between 0.27 and 0.39 and two samples, clearly identifiable in the PCA plots, whose genomes were ca. 90% of pig origin. Our results confirm that for these populations, hybridization with the domestic counterpart is an ongoing process, possibly strongly related to pig farming and wild boar management practices. The observation of almost pure pigs within the wild boar sample suggests the presence of released or escaped animals which became feral. Future research, ideally comprising additional samples from Eastern Europe and the Balkan area, could help identify the possible existence of broader geographical patterns relevant for evolution and conservation management, and the potential influence of the different breeding systems established in Eastern and Western Europe. For instance, whereas in Germany and the Netherlands industrial pig farms are common^44^, in Bulgaria the traditional breeding system is associated with high bidirectional hybridization^45^.

Additional analyses targeting the introgressed chromosomal regions, the genes within those regions and the processes controlled by them, are needed to provide insights in the biological and potential evolutionary consequences of hybridization. Introgression from pigs could lead to maladaptation^20^, but it could also benefit the hybrids^21^, e.g. by increasing the species’ reproductive rates^6,23^. Understanding which are the inherited chromosomal regions and whether there is selection after introgression would provide important insights for the management of the species. Europe is currently facing a widespread demographic increase^5^. Such a trend has been observed both in countries where no hybrids were detected (e.g. Baltic countries) and countries with high percentage of hybrids (e.g. Serbia), suggesting a minor role of hybridization in increasing the species invasiveness. However, it would be interesting to simulate different demographic scenarios for populations with variable levels of genetic admixture to allow the refinement of management policies. Additionally, the presence of recent admixture raises concern regarding the potential risk for the spread of pig-borne diseases. This is particularly important considering the introduction of African Swine Fever in the Caucasus^46^ and, later on, its spread into the European Union^10^. While enhanced biosecurity could prevent the contagion in farmed animals, the risk remains high for free-ranging pigs, and contact with wild boar could be a potential route of infection^10^. This risk would be even higher if young individuals (0.5–2 years) are involved in the admixture event and the infection, as they show higher connectivity with other individuals within the population compared to older animals^47^. Additionally, the increasing number of wild boar in urban areas^48^ could favour the transmission of zoonoses and other diseases and could be potentially aggravated by the introgression, though hybridization, of tameness traits.

Unfortunately, as highlighted by the presence of almost pure pigs in the wild boar sample, the identification of hybrids in the field can be problematic even for experienced personnel, as reported for other species^49^, highlighting the importance of genetic studies and the selection of reference populations. However, considering that the current wild boar population represents a continuum of genotypes, it is probably unfeasible to reduce the hybridization levels by removal of admixed individuals. Accordingly, there is an urgent need to develop strategies to reduce hybridization and its underlying causes. Our results show the importance of long term genetic monitoring of populations. Considering that hybridization can change over time, analytical approaches might have different resolution power and natural movement of animals might reshape temporal population substructure. Furthermore, we suggest to implement strict genetic controls on source animals for release practices, reducing the use of farmed animals, which have been shown^15,17^ to be one of the main sources of introgression. Additionally, SNP chips allow for animal traceability and they could be used to detect illegal introductions. At the same time, efforts should be made to increase public awareness of the risks associated with illegal introductions, and to improve biosecurity in free-ranging pig farms. Furthermore, our results highlight the internal substructure of the European wild boar population. This underlines the need to develop management plans that will account for regional differences (e.g. France or the Netherlands) and facilitate implementation of cross border strategies (e.g. Balkans and Carpathians). A combination of such measures will contribute to reducing the contact rates between wild boar and pigs, decreasing the occurrence of both hybridization events and the risk of disease spread.

## Electronic supplementary material

Supplementary information

## Data Availability

The datasets generated and analysed during the current study are available from the corresponding author on request.
