# Author Correction: Hotspots of recent hybridization between pigs and wild boars in Europe

**DOI:** 10.1038/s41598-019-55961-7

**Published:** 2019-12-24

**Authors:** Laura Iacolina, Cino Pertoldi, Marcel Amills, Szilvia Kusza, Hendrik-Jan Megens, Valentin Adrian Bâlteanu, Jana Bakan, Vlatka Cubric-Curik, Ragne Oja, Urmas Saarma, Massimo Scandura, Nikica Šprem, Astrid Vik Stronen

**Affiliations:** 1 Department of Chemistry and Bioscience, Aalborg University, Frederik Bajers Vej 7H, 9220 Aalborg, Denmark; 2 Aalborg Zoo, Mølleparkvej 63, 9000 Aalborg, Denmark; 3 Centre for Research in Agricultural Genomics (CRAG), CSIC-IRTA-UAB-UB, Campus de la Universitat Autònoma de Barcelona, Bellaterra, 08193 Spain; 4 Departament de Ciència Animal i dels Aliments, Universitat Autònoma de Barcelona, Bellaterra, 08193 Spain; 5 Animal Genetics Laboratory, Faculty of Agricultural and Food Sciences and Environmental Management, University of Debrecen, Böszörményi 138, 4032 Debrecen, Hungary; 6 Wageningen University & Research, Animal Breeding and Genomics, Droevendaalsesteeg 1, Wageningen, 6708PD The Netherlands; 7 Institute of Life Sciences, Faculty of Animal Science and Biotechnologies, University of Agricultural Sciences and Veterinary Medicine, Calea Mănăştur 3-5, 400372 Cluj-Napoca, Romania; 8 Technical University of Zvolen, Department of Phytology, Ul. T. G. Masaryka 24, 96053 Zvolen, Slovakia; 9 Department of Animal Science, Faculty of Agriculture, University of Zagreb, Svetošimunska cesta 25, 10000 Zagreb, Croatia; 10 Department of Zoology, Institute of Ecology and Earth Sciences, University of Tartu, Vanemuise 46, 51003 Tartu, Estonia; 11 Department of Veterinary Medicine, University of Sassari, via Muroni 25, I-07100 Sassari, Italy; 12 Department of Fisheries, Beekeeping, Game Management and Special Zoology, Faculty of Agriculture, University of Zagreb, Svetošimunska cesta 25, 10000 Zagreb, Croatia; 13 Department of Biology, Biotechnical Faculty, University of Ljubljana, Večna pot 111, 1000 Ljubljana, Slovenia

Correction to: *Scientific Reports* 10.1038/s41598-018-35865-8, published online 26 November 2018

The original version of this Article contained a typographical error in the spelling of the author Vlatka Cubric-Curik, which was incorrectly given as Vlatka Cubric-Curic. This has now been corrected in the PDF and HTML versions of the Article, and in the accompanying Supplementary Information file.

