# Supplementary material for: Hotspots of recent hybridization between pigs and wild boars in Europe
Source: Sci Rep. 2018 Nov 26;8:17372. doi: 10.1038/s41598-018-35865-8 (PMC6255867; doi:10.1038/s41598-018-35865-8)

**Title: Hotspots of recent hybridization between pigs and wild boars in Europe**

Laura Iacolina, Cino Pertoldi, Marcel Amills, Szilvia Kusza, Hendrik-Jan Megens, Valentin Adrian Bâlteanu, Jana Bakan, Vlatka Cubric-Curik, Ragne Oja, Urmas Saarma, Massimo Scandura, Nikica Šprem, Astrid Vik Stronen

**Supplementary Table S1.** Animals analysed for this study. a) wild boar (WB); b) domestic pig (DP). Population = Country (for WB)/breed (for DP) of origin of the animals, N = number of genotypes, Cluster = cluster the population was assigned to for some of the analysis.

a)

| **Population** | **Population abbreviation** | **N** | **Cluster** | **Cluster abbreviation** |
| --- | --- | --- | --- | --- |
| Bosnia | Bos | 13 | Balkans | WB-Bal |
| Bulgaria | Bul | 5 | Balkans | WB-Bal |
| Croatia | Cro | 15 | Balkans | WB-Bal |
| Greece | Gre | 8 | Balkans | WB-Bal |
| Serbia | Ser | 14 | Balkans | WB-Bal |
| Slovenia | Slv | 12 | Balkans | WB-Bal |
| Hungary | Hun | 14 | Carpathians | WB-Car |
| Romania | Rom | 18 | Carpathians | WB-Car |
| Slovakia | Slk | 5 | Carpathians | WB-Car |
| Portugal | Por | 9 | Iberia | WB-Ibe |
| Spain | Spa | 14 | Iberia | WB-Ibe |
| Italy | Ita | 19 | Italy | WB-Ibe |
| Sardinia | Sar | 25 | Sardinia | WB-Ibe |
| Austria | Aus | 9 | Central-West Europe | WB-CW |
| Belgium | Bel | 6 | Central-West Europe | WB-CW |
| France | Fra | 25 | Central-West Europe | WB-CW |
| Germany | Ger | 16 | Central-West Europe | WB-CW |
| Luxembourg | Lux | 4 | Central-West Europe | WB-CW |
| Netherlands | Ned | 25 | Central-West Europe | WB-CW |
| Estonia | Est | 15 | Central-North-Eastern Europe | WB-CNE |
| Finland | Fin | 3 | Central-North-Eastern Europe | WB-CNE |
| Poland | Pol | 12 | Central-North-Eastern Europe | WB-CNE |
| Russia | Rus | 4 | Central-North-Eastern Europe | WB-CNE |
| Armenia | Arm | 3 | Near Eastern | WB-NE |
| Iran | Ira | 5 | Near Eastern | WB-NE |
| Turkey | Tur | 8 | Near Eastern | WB-NE |
| **TOT** |  | **306** |  |  |

b)

| **Population** | **Population abbreviation** | **N** | **Country** | **Cluster** | **Cluster abbreviation** |
| --- | --- | --- | --- | --- | --- |
| Black Slavonian | BS | 10 | Croatia | Balkans | DP-Bal |
| Turopolje | TU | 13 | Croatia | Balkans | DP-Bal |
| Mangaliza | MA | 25 | cross-country | Carpathians | DP-Car |
| Poland domestic | PO | 8 | Poland | Central-East Europe | DP-CE |
| Pulawska Spot | PU | 11 | Poland | Central-East Europe | DP-CE |
| Estonia domestic | ES | 12 | Estonia | Central-Nord Europe | DP-CN |
| Linderodssvin | LI | 10 | Sweden | Central-Nord Europe | DP-CN |
| Berkshire | BK | 8 | - | Commercial | DP-Com |
| Duroc | DU | 10 | - | Commercial | DP-Com |
| Large White | LW | 7 | - | Commercial | DP-Com |
| Pietrain | PI | 10 | - | Commercial | DP-Com |
| Yorkshire | YO | 9 | - | Commercial | DP-Com |
| Angler Sattleschwein | AS | 9 | Germany | Central-West Europe | DP-CW |
| Bunte Bentheimer | BB | 11 | Germany | Central-West Europe | DP-CW |
| Presticke | PR | 15 | Czech Republic | Central-West Europe | DP-CW |
| Bisaro | BI | 11 | Portugal | Iberia | DP-Ibe |
| Cato Murciano | CM | 25 | Spain | Iberia | DP-Ibe |
| Iberian | IB | 19 | Spain | Iberia | DP-Ibe |
| Manchado de Jabugo | MJ | 6 | Spain | Iberia | DP-Ibe |
| Negra Iberica | NI | 13 | Spain | Iberia | DP-Ibe |
| Retinto | RE | 9 | Spain | Iberia | DP-Ibe |
| Calabrese | CA | 10 | Italy | Italy | DP-Ita |
| Casertana | CT | 7 | Italy | Italy | DP-Ita |
| Cinta Senese | CS | 8 | Italy | Italy | DP-Ita |
| Mora Romagnola | MR | 8 | Italy | Italy | DP-Ita |
| Nera Siciliana | NS | 7 | Italy | Italy | DP-Ita |
| Sarda | SA | 8 | Italy (Sardinia) | Italy | DP-Ita |
| **TOT** |  | **299** |  |  |  |

**Supplementary Table S2.** Maximum K values used for Admixture analysis and K value identified by cross-validation (CV) method.

| **Dataset** | **30K** | **1K** | **30K-CV** | **1K-CV** |
| --- | --- | --- | --- | --- |
| All | 30 | 30 | 27 | 27 |
| Wild boar | 20 | 20 | 12 | 12 |
| Balkans | 15 | 15 | 10 | 9 |
| Carpathians | 10 | 15 | 9 | 11 |
| Central Europe | 20 | 20 | 14 | 15 |
| Iberia | 15 | 20 | 10 | 12 |
| Italy | 15 | 20 | 13 | 12 |
| Northern Europe | 10 | 15 | 8 | 9 |
| Sardinia | 10 | 15 | 9 | 8 |

**Supplementary Table S3.** Pairwise F_ST_ values, all comparisons were significant (p < 0.05). Abbreviations are the same as in Table S1.

|  | **DP-Com** | **DP-CN** | **DP-CE** | **DP-CW** | **DP-Ita** | **DP-Ibe** | **DP-Car** | **DP-Bal** | **WB-Sar** | **WB-Ita** | **WB-CW** | **CNE-WB** | **WB-Ibe** | **WB-Car** | **WB-Bal** | **WB-NE** |
| --- | --- | --- | --- | --- | --- | --- | --- | --- | --- | --- | --- | --- | --- | --- | --- | --- |
| **DP-Com** | - |  |  |  |  |  |  |  |  |  |  |  |  |  |  |  |
| **DP-CN** | 0.053 | - |  |  |  |  |  |  |  |  |  |  |  |  |  |  |
| **DP-CE** | 0.062 | 0.084 | - |  |  |  |  |  |  |  |  |  |  |  |  |  |
| **DP-CW** | 0.045 | 0.064 | 0.074 | - |  |  |  |  |  |  |  |  |  |  |  |  |
| **DP-Ita** | 0.070 | 0.086 | 0.108 | 0.078 | - |  |  |  |  |  |  |  |  |  |  |  |
| **DP-Ibe** | 0.063 | 0.081 | 0.099 | 0.070 | 0.056 | - |  |  |  |  |  |  |  |  |  |  |
| **DP-Car** | 0.096 | 0.113 | 0.134 | 0.100 | 0.077 | 0.070 | - |  |  |  |  |  |  |  |  |  |
| **DP-Bal** | 0.113 | 0.132 | 0.152 | 0.117 | 0.105 | 0.097 | 0.112 | - |  |  |  |  |  |  |  |  |
| **WB-Sar** | 0.193 | 0.216 | 0.241 | 0.200 | 0.165 | 0.161 | 0.161 | 0.220 | - |  |  |  |  |  |  |  |
| **WB-Ita** | 0.164 | 0.182 | 0.208 | 0.171 | 0.140 | 0.135 | 0.123 | 0.186 | 0.124 | - |  |  |  |  |  |  |
| **WB-CW** | 0.144 | 0.158 | 0.179 | 0.147 | 0.118 | 0.111 | 0.090 | 0.155 | 0.137 | 0.088 | - |  |  |  |  |  |
| **CNE-WB** | 0.149 | 0.165 | 0.189 | 0.154 | 0.129 | 0.122 | 0.102 | 0.166 | 0.143 | 0.087 | 0.053 | - |  |  |  |  |
| **WB-Ibe** | 0.156 | 0.175 | 0.200 | 0.162 | 0.138 | 0.127 | 0.123 | 0.183 | 0.175 | 0.127 | 0.074 | 0.100 | - |  |  |  |
| **WB-Car** | 0.144 | 0.160 | 0.183 | 0.148 | 0.126 | 0.121 | 0.101 | 0.163 | 0.143 | 0.083 | 0.068 | 0.036 | 0.113 | - |  |  |
| **WB-Bal** | 0.148 | 0.163 | 0.184 | 0.151 | 0.123 | 0.119 | 0.096 | 0.158 | 0.135 | 0.071 | 0.065 | 0.042 | 0.110 | 0.018 | - |  |
| **WB-NE** | 0.224 | 0.245 | 0.275 | 0.236 | 0.234 | 0.227 | 0.232 | 0.277 | 0.307 | 0.273 | 0.235 | 0.211 | 0.303 | 0.194 | 0.186 | - |

**Supplementary Figure S1.** Admixture plots of European wild boar and domestic pigs based on 30K SNPs. Plots represent (a) Central Europe (K=14), (b) Iberia (K=10), (c) Italy (K=13), (d) Sardinia (K=9), (e) Balkans (K=10), (f) Carpathians (K=9) and (g) Northern Europe (K=8). Abbreviations correspond to Population abbreviation in Table S1.

a)
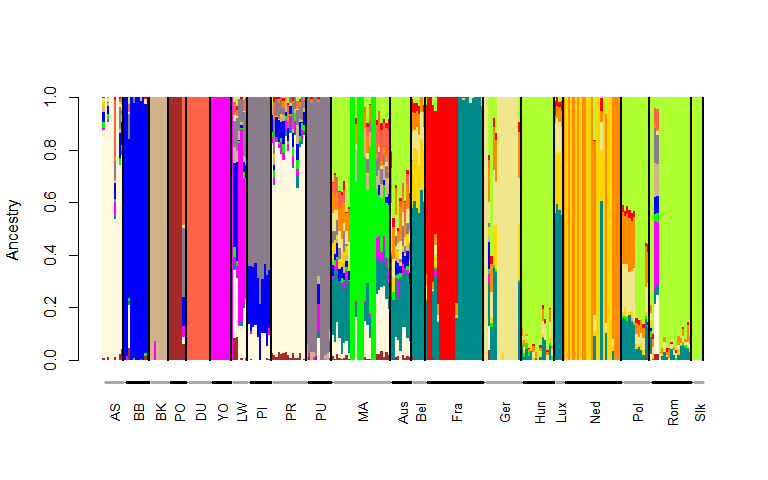
b)
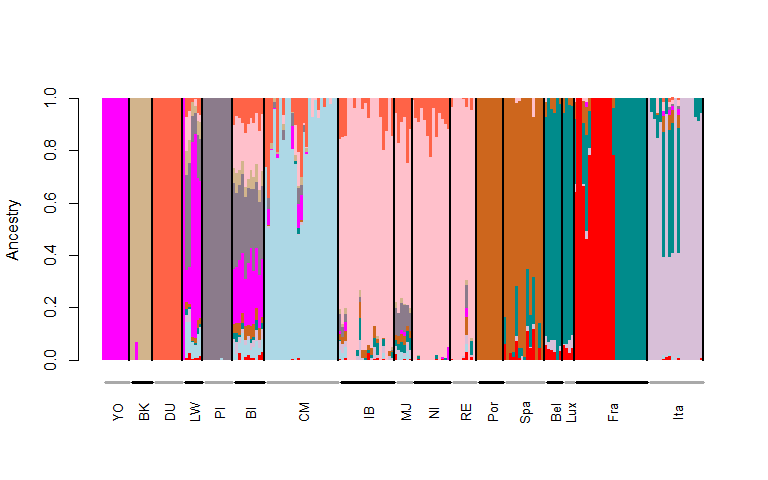


c)
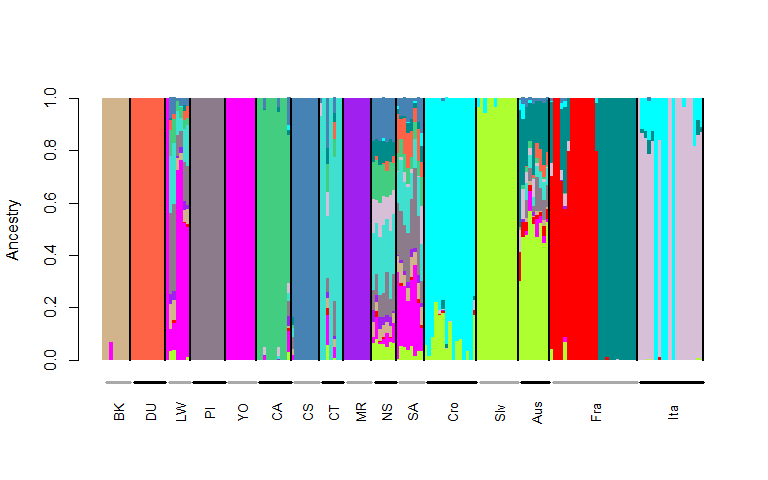
d)
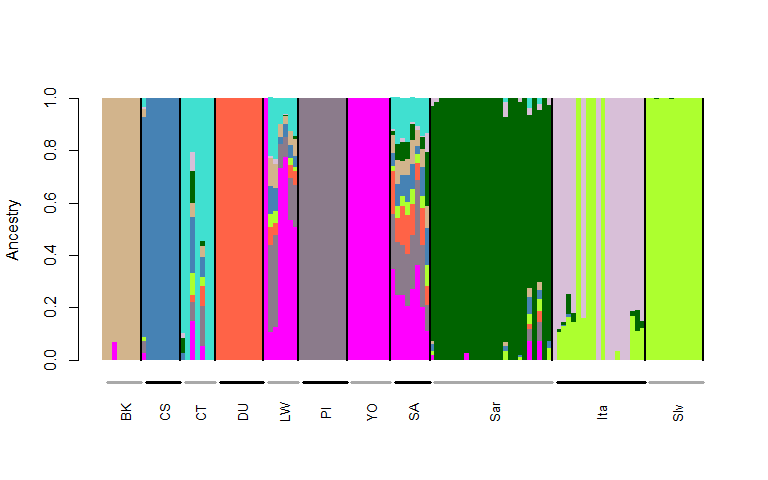
e)
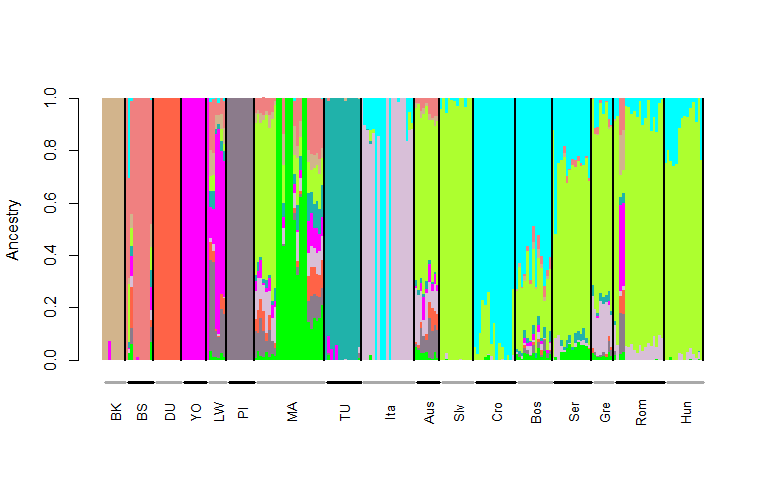


f)
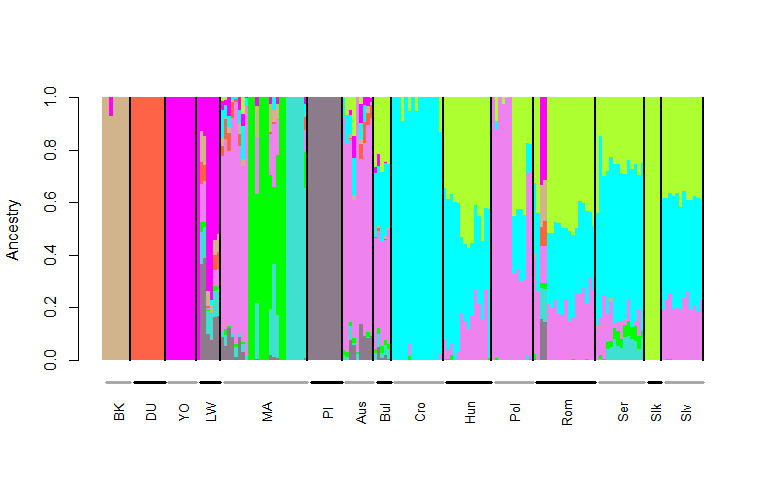


g)
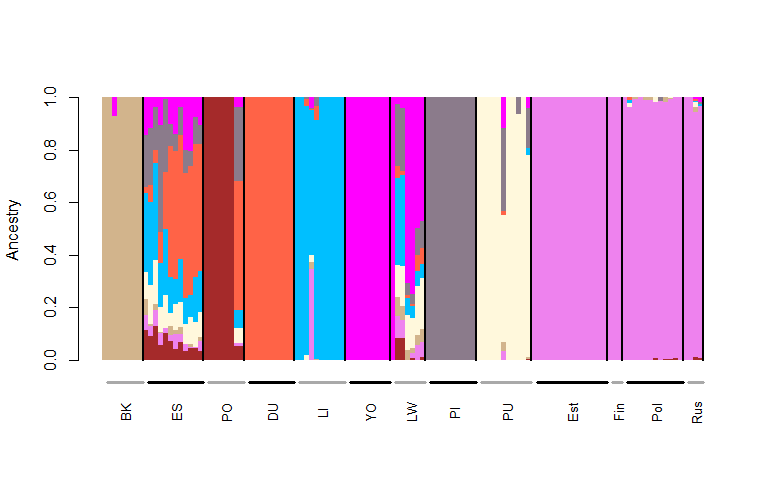


**Supplementary Figure S2.** Admixture plots of European wild boar and domestic pigs for K 2-4. Plots represent: (a) K=2 with 30K SNPs, (b) K=2 with 1K SNPs, (c) K=3 with 30K SNPs, (d) K=3 with 1K SNPs, (e) K=4 with 30K SNPs, (f) K=4 with 1K SNPs. Abbreviations correspond to Cluster abbreviation in Table S1.

(a)
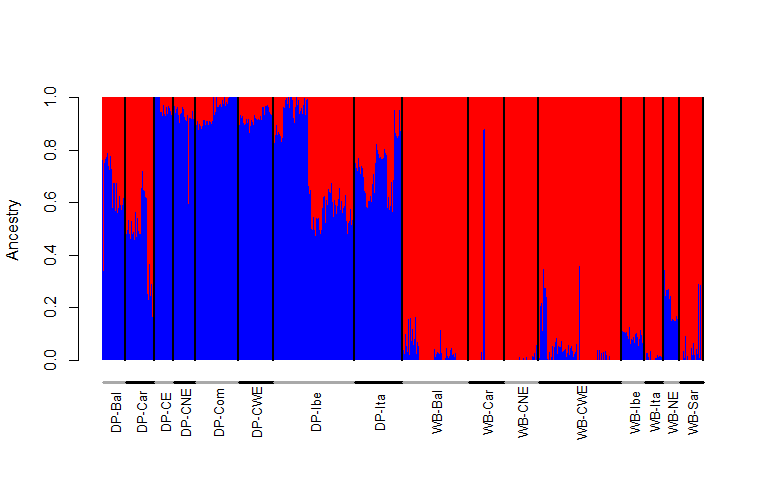
(b)
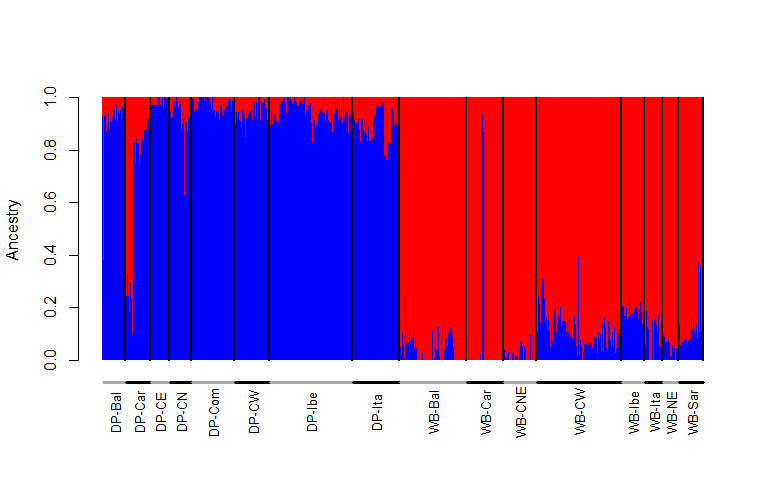


(c)
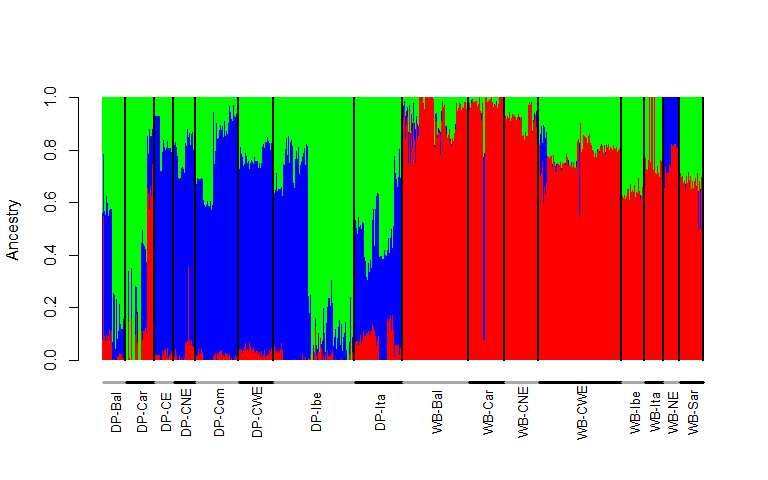
(d)
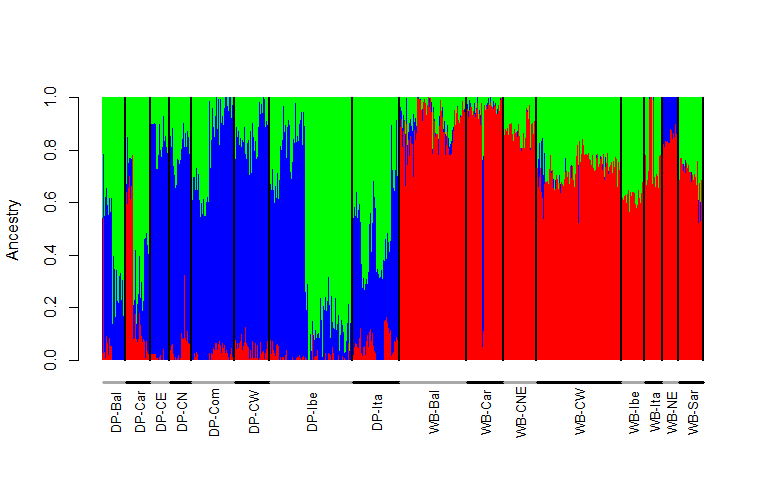


(e)
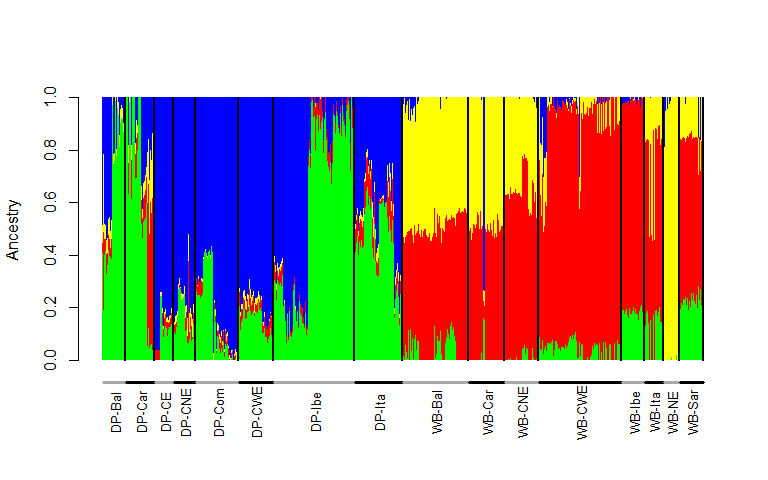
(f)
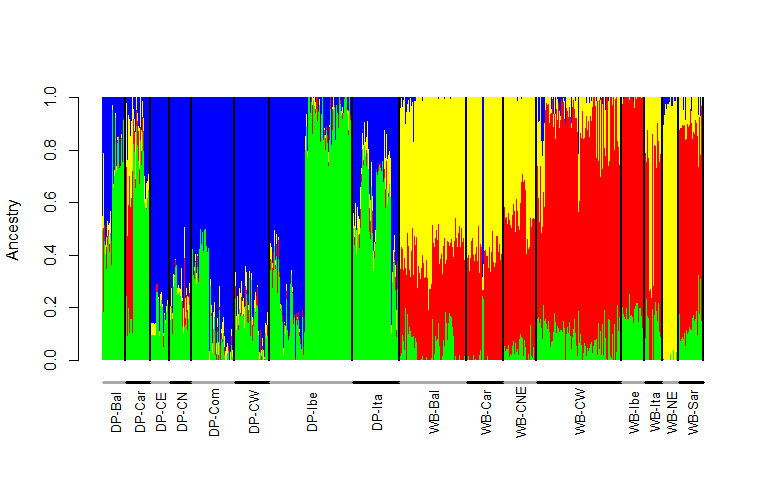


**Supplementary Figure S3.** Admixture plots of European wild boar and domestic pigs based on 1K SNPs. Plots represent (a) Central Europe (K=15), (b) Iberia (K=12), (c) Italy (K=12), (d) Sardinia (K=8), (e) Balkans (K=9), (f) Carpathians (K=11) and (g) Northern Europe (K=9). Abbreviations correspond to Population abbreviation in Table S1.

a)
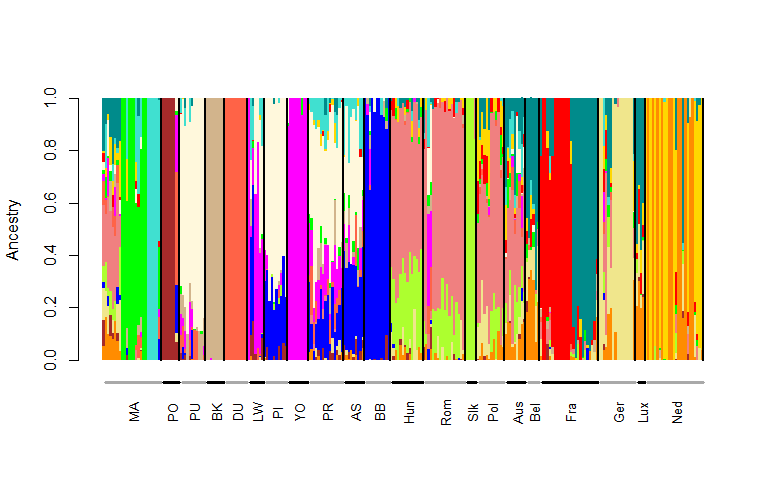
b)
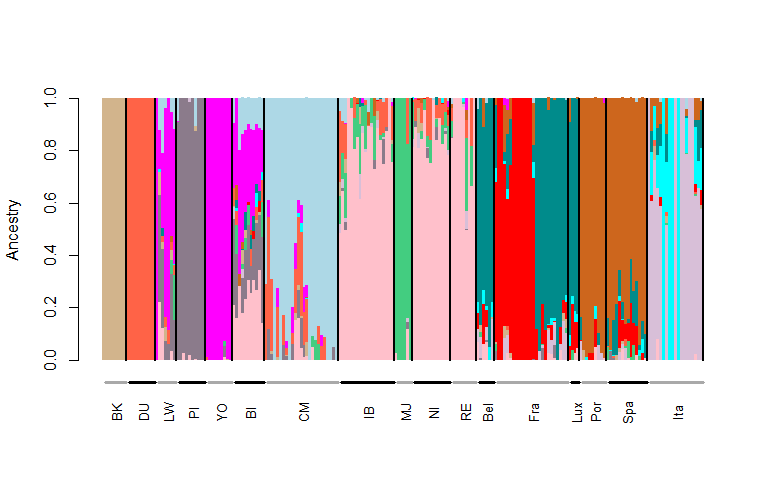


c)
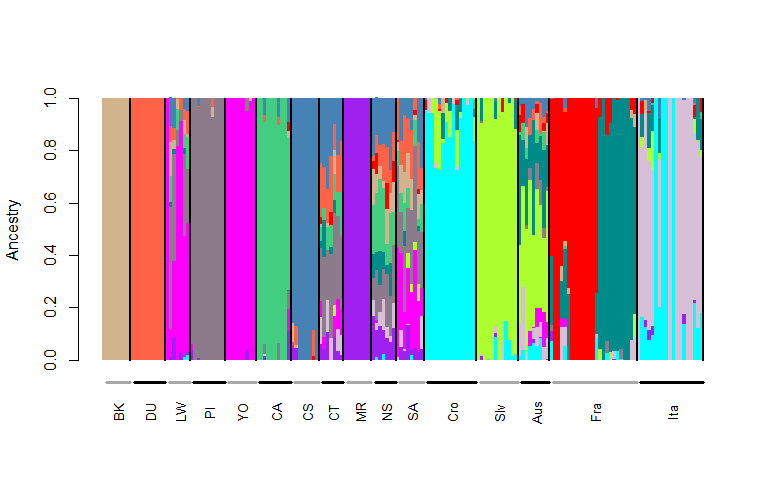
d)
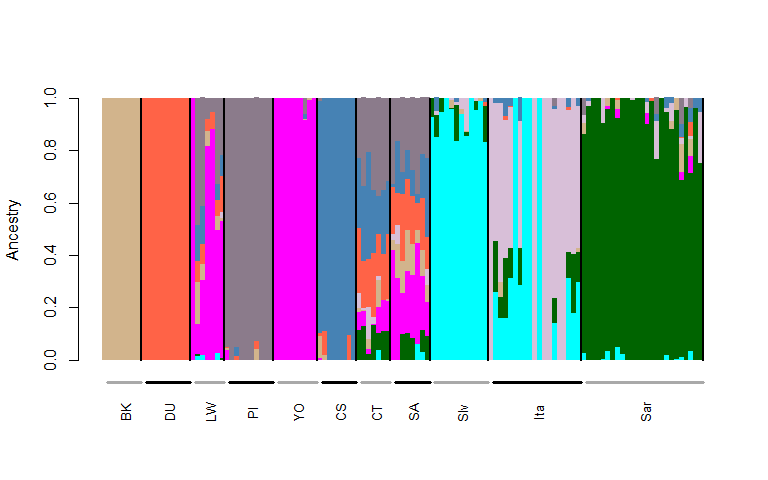
e)
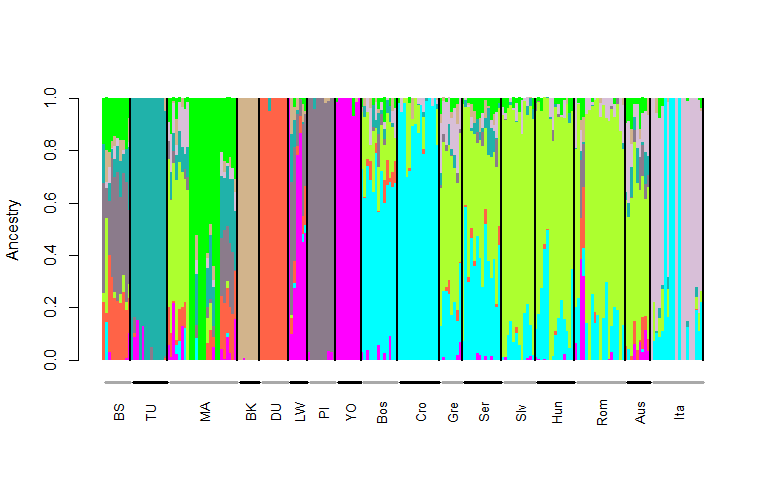
f)
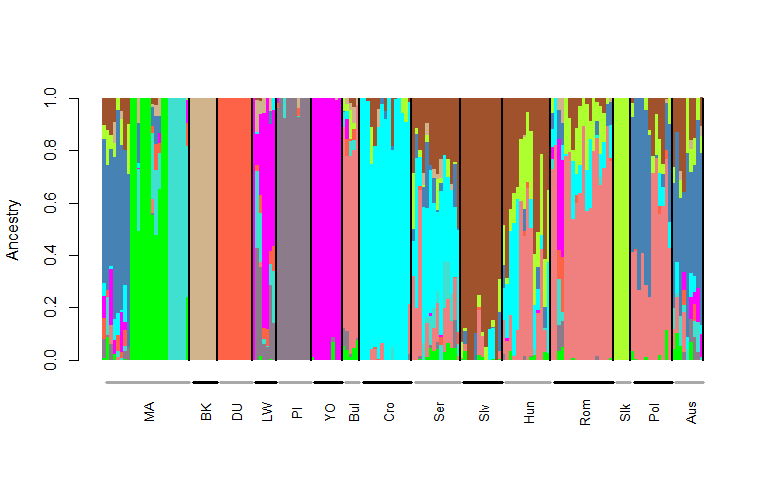
g)
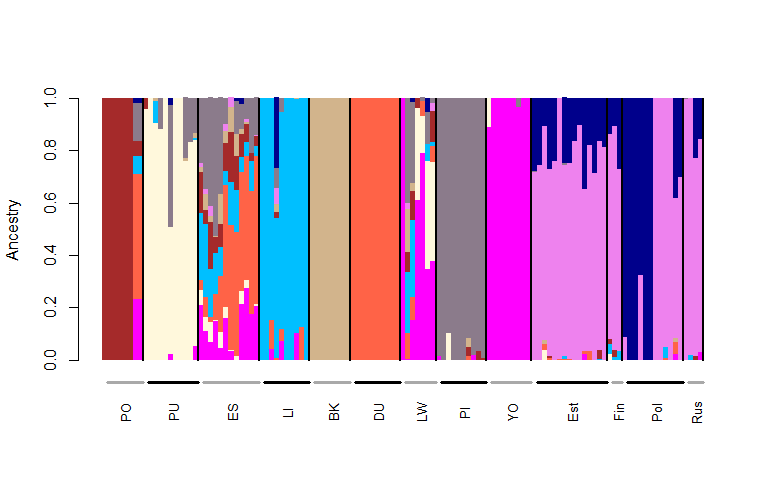

Supplement: Supplementary file 1 — Supplementary information [file 41598_2018_35865_MOESM1_ESM.docx]
